# Efficacy of Apremilast Gels in Mouse Model of Imiquimod-Induced Psoriasis Skin Inflammation

**DOI:** 10.3390/pharmaceutics15102403

**Published:** 2023-09-29

**Authors:** Marcelle Silva-Abreu, Lilian Sosa, Lupe Carolina Espinoza, María-José Fábrega, María J. Rodríguez-Lagunas, Mireia Mallandrich, Ana Cristina Calpena, María Luisa Garduño-Ramírez, María Rincón

**Affiliations:** 1 Departament de Farmàcia, Tecnologia Farmacèutica, i Fisicoquímica, Facultat de Farmàcia i Ciències de l’Alimentació, Universitat de Barcelona (UB), 08028 Barcelona, Spain; mireia.mallandrich@ub.edu (M.M.); anacalpena@ub.edu (A.C.C.); 2 Institut de Nanociència i Nanotecnologia IN2UB, University of Barcelona, 08028 Barcelona, Spain; lcespinoza@utpl.edu.ec (L.C.E.); m.rincon@ub.edu (M.R.); 3 Research Institute of Applied Sciences and Technology, National Autonomous University of Honduras (UNAH), Tegucigalpa 11101, Honduras; lilian.sosa@unah.edu.hn; 4 Microbiology Research Institute, National Autonomous University of Honduras (UNAH), Tegucigalpa 11101, Honduras; 5 Departamento de Química y Ciencias Exactas, Universidad Técnica Particular de Loja, Loja 1101608, Ecuador; 6 Department of Experimental and Health Sciences, Parc de Recerca Biomèdica de Barcelona, University Pompeu Fabra (UPF), 08005 Barcelona, Spain; mjfabrega.f@gmail.com; 7 Department of Biochemistry and Physiology, Faculty of Pharmacy and Food Sciences, University of Barcelona, 08028 Barcelona, Spain; mjrodriguez@ub.edu; 8 Nutrition and Food Safety Research Institute (INSA-UB), 08921 Barcelona, Spain; 9 Center for Chemical Research, Institute for Research Basic and Applied Sciences, Autonomous University of the State of Morelos, Av. Universidad 1001, Cuernavaca 62209, Mexico; lgarduno@uaem.mx; 10 Departament de Ciència de Materials i Química Física, Facultat de Química, Universitat de Barcelona (UB), C. Martí i Franquès 1-11, 08028 Barcelona, Spain

**Keywords:** apremilast, imiquimod, topical psoriasis, skin formulations, Pluronic F127, Sepigel 305^®^, Carbomer 940

## Abstract

Apremilast (APM) is a novel drug for the treatment of psoriasis and psoriatic arthritis. APM is a phosphodiesterase 4 (PDE4) inhibitor, raising intracellular cAMP levels and thereby decreasing the inflammatory response by modulating the expression of TNF-α, IL-17, IL-23, and other inflammatory cytokines. The goal of this study is to develop APM gels as a new pharmaceutical formulation for the treatment of topical psoriasis. APM was solubilized in Transcutol-P and incorporated into Pluronic F127, Sepigel, and carbomer bases at different proportions. All formulations were characterized physiochemically. A biopharmaceutical study (release profile) was performed, and ex vivo permeation was evaluated using a human skin model. A toxicity assay was carried out on the HaCaT cell line. A mouse model of imiquimod-induced psoriasis skin inflammation was carried out to determine its efficacy by histological analysis, RNA extraction, and RT-qPCR assays. APM gel formulations showed good physicochemical characteristics and a sustained release profile. There was no permeation of any gel measured through human skin, indicating a high retained amount of APM on the skin. Cell viability was greater than 80% at most dilution concentrations. APM gels treated the psoriasis mouse model, and it shows a reduction in the proinflammatory cytokines (IL-8, IL-17A, IL-17F, and IL-23). APM gels could be a new approach for the treatment of topical psoriasis.

## 1. Introduction

Psoriasis is an autoimmune, recurrent inflammatory skin disease with an estimated incidence of 2% of the world’s population [1,2] characterized by infiltration of inflammatory cells in the epidermis with severe alterations in the dermal vascular system, as well as the development of erythematous, scaly, pruritic, indurated, and often painful skin plaques [3,4,5]. Psoriasis pathogenesis is handled by proinflammatory cytokines, and psoriasis relates to an increased risk for comorbidities, including, but not limited to, psoriatic arthritis, diabetes mellitus, cardiovascular disease, obesity, inflammatory bowel disease, and nonalcoholic fatty liver disease, compared with the common population. A review of available advances in understanding the inflammatory nature of psoriasis shows that research efforts have focused on elucidating the roles of specific proinflammatory cytokines that contribute to disease pathogenesis, with the aim of developing novel targeted therapies [6,7]. Current therapies used to control psoriasis encompass a relatively wide range of medications, including topical steroids, vitamin D derivatives, phytochemicals (aloe vera, *Datura metel* L. flowers, and Curcuma longa), phototherapy, systemic biological drugs (secukinumab, adalimumab, and ustekinumab), and non-biological drugs (anti-inflammatory drugs, methotrexate, and cyclosporine) [8,9].

Apremilast (APM) is a selective inhibitor of (PDE)-4 used for psoriatic arthritis and moderate to severe plaque psoriasis administered orally [10,11]. The drug’s mode of operation involves the modulation of a series of proinflammatory and anti-inflammatory mediators (TNFα, IL-23, IL-10, and IL-17). This modulation is accomplished by elevating intracellular levels of cyclic adenosine monophosphate [11,12]. APM is considered a moderately safe drug with some serious adverse effects. Common adverse effects include diarrhea, nausea, upper respiratory tract infection, nasopharyngitis, and headache. Due to the drug acting on many cell types and being quite well tolerated, investigators have explored its efficacy in chronic inflammatory diseases that are frequently resistant to therapy. Some of these include atopic dermatitis, Behçet’s disease, hidradenitis suppurativa, alopecia areata, other variants of psoriasis, cutaneous sarcoidosis, and discoid lupus [13]. Some studies have reported promising results of different APM topical formulations, including microemulsions, nanoemulgels, nanostructured lipid carriers, and nanocrystals [10,14,15,16].

Topical therapy is typically regarded as the initial treatment option and is generally effective in addressing plaque psoriasis and milder forms of psoriasis that affect up to 25% of the body surface area [5]. Transdermal delivery provides a cutting edge over injectables and oral routes by increasing patient compliance and avoiding first-pass metabolism. Transdermal delivery not only provides controlled administration of the drug, but also allows continuous input of drugs with short biological half-lives and eliminates pulsed entry into the systemic circulation, which often causes undesirable side effects. Hence, the transdermal system for drug delivery provides the advantages of elimination of hepatic first-pass metabolism, enhancement of therapeutic efficiency, and maintenance of a steady plasma level of the drug [17].

In topical psoriasis therapy, diverse vehicles (creams, solutions, ointments, gels, or foams) could be used. They show different effectiveness, and patients have different preferences regarding the formulation texture and organoleptic properties. A semi-solid texture is typically favored for applying treatments to the body, whereas liquid formulations are primarily used on the scalp. Patients with skin conditions generally prefer gels or creams over sticky and oily ointments, as gels are versatile and suitable for both body and scalp applications [18].

Regarding polymers for preparing gels, Pluronic^®^ identifies a generic block copolymer of two units of poly(ethylene glycol), separated by a central poly(propylene oxide) unit. One specific type is Pluronic F127, which is very popular due to its low toxicity and high biocompatibility [19]. Also, Sepigel 305^®^ is another type of polymer that is composed of (i) the fatty oil isoparaffin, (ii) the gelation-promoting polymer polyacrylamide, and (iii) the non-ionic emulsifier laureth-7. Sepigel 305^®^ forms hydrogels with the advantage of allowing the addition of hydrophilic and lipophilic substances [20]. Additionally, there is the polymer carbomer, which has been extensively used as the main active ingredient carrier for transdermal applications. It offers several advantages, including high viscosity, excellent compatibility, good stability, and excellent tissue compatibility [21,22,23]. Numerous functional polysaccharides are available, capable of serving as film formers, gelling agents, thickeners, conditioners, and emulsifiers, such as hyaluronic acid, which is applied as a moisturizing and bioactive ingredient [24,25].

This work aimed to develop and evaluate semi-solid formulations containing APM to manage psoriasis using a topical therapy approach since there are no topical commercial products with APM on the market. The formulations were fully characterized and evaluated for their efficacy and tolerability. To this end, the formulations were tested on mice with imiquimod-induced psoriasis. Histological analysis of the skin was conducted, and PCR assays were performed to assess the inflammatory cytokines. The tolerability of the formulations was evaluated on HaCaT cells, determining their cell viability after being exposed to the formulations. Additionally, blank formulations were also tested on skin-healthy volunteers, monitoring changes in transepidermal water loss, a parameter that shows a good correlation with skin barrier integrity.

## 2. Materials and Methods

### 2.1. Materials

APM (460.5 Da, molecular weight, and 99.6% purity) was acquired from Wuhan Senwayer Century Chemical (Wuhan, China). Carbomer 940 (CAS 9003-01-4) and Pluronic F127 (CAS 9003-11-6) were obtained from Fagron (Waregem, Belgium). Hyaluronic acid (HA) with a molecular weight of 50 kDa was purchased from Cn Lab Canada, Asian Group (Xi’an, China). Sepigel 305^®^ (CAS 64742-47-8) (Polyacrylamide, C13-14 Isoparaffin Laureth-7) was obtained from SEPPIC. Diethylene glycol monoethyl ether (Transcutol-P) was provided by Gattefossé (Saint-Priest, France). Phosphate-buffered saline (PBS) tablets were purchased from Sigma (Darmstadt, Germany), processed according to the manufacturer’s instructions, and stored in the refrigerator for further use. The HaCaT cell line was acquired from Cell Line Services (Eppelheim, Germany), and reagents utilized for cell culture were purchased from Gibco (Carcavelos, Portugal). The reagents utilized for the MTT assay were purchased from Invitrogen Alfagene^®^ (Carcavelos, Portugal). Reagents used for the MTT assay were purchased from Invitrogen Alfagene^®^ (Carcavelos, Portugal). All the reagents for histological procedures were purchased from Sigma and Thermo Fisher Scientific (Barcelona, Spain). Milli-Q^®^ purified water was used in all experiments. To conclude, all reagents used in this study were of analytical grade.

### 2.2. Methods

#### 2.2.1. APM Gel Preparations: Carbopol, Pluronic, and Sepigel

Previously, a solution of APM was prepared by dissolving the drug into different concentrations of Transcutol-P (20%, 30%, and 40%, *w*/*v*) and stirring at 700 rpm until drug dissolution in order to optimize the solvent concentration for the final formulations. Afterwards, different gel bases (Carbomer 940 (Carb), Pluronic (Plur), and Sepigel 305^®^ (Sepi)) were prepared and incorporated into the solution. The optimized formulation for each gel is described below:

APM-Plur: APM (0.15%) was added to 30% (*w*/*v*) of Transcutol-P and stirred at 700 rpm until drug dissolution. After that, 0.2% (*w*/*v*) of HA and a q.s.p. of water were added to the previous solution. The gel formed after adding 20% (*w*/*v*) pluronic. The APM-Plur gel was manually homogenized for 30 min. This gel shows a thermoreversible profile at 25 °C (gel) and 2–8 °C (liquid).

APM-Carb: The gel was formulated using Carbomer 940 (2% *w*/*v*), which was dispersed in Milli-Q water and hydrated for 6 h (gel base). After that, NaOH 1N was added to alkalinize the formulation and achieve polymer gelation. Separately, APM (0.15%) was added to 30% (*w*/*v*) of Transcutol-P and stirred at 700 rpm until drug dissolution. This phase was added to the gel base plus 0.2% (*w*/*v*) of HA. The APM-Carb gel was manually homogenized for 30 min.

APM-Sepi: APM (0.15%) was added to 30% (*w*/*v*) of Transcutol-P and stirred at 700 rpm until drug dissolution. After that, 0.2% (*w*/*v*) of HA and Sepigel 305^®^ (2% *w*/*v*) were dispersed in Milli-Q water q.s.p. into the previous solution. The APM-Sepi gel was manually homogenized for 30 min.

Following preparation, the gels were left unstirred until they reached equilibrium for one day at 25 °C (room temperature) before being used.

#### 2.2.2. Physicochemical Characterization

##### Swelling and Degradation Studies

A gravimetric method was used to obtain the swelling ratio (SR) and test degradation, represented as the percentage of weight loss (WL). Dried and fresh APM-Plur, APM-Carb, and APM-Sepi were used for the swelling and degradation tests, respectively. In both experiments, APM-Plur, APM-Carb, and APM-Sepi were incubated in PBS solution (pH = 5.5) at 32 °C for 30 min for APM-Plur and APM-Carb and for 20 min for APM-Sepi gel (n = 3). The samples were removed, and their weight was measured after blotting away surface water at the specific time. The SR was estimated using the following equation and expressed by kinetic modeling:

(1) $$\mathrm{SR}=\frac{WS-Wd}{Wd}$$

*Ws* is the weight of the swollen APM-Plur and APM-Carb gels at 5-min intervals for 30 min, and *Wd* is the weight of the dried gel. While for APM-Sepi, the time for the weight of the swollen gel was at 3 min intervals for 20 min.

WL was calculated in following the equation and expressed by kinetic modeling:

(2) $$\mathrm{WL}\;(\%)=\frac{Wi-Wd}{Wi}\times 100$$

*Wi* is the initial weight of APM-Plur and APM-Carb, and *Wd* is the gel weight each 5 min. While for APM-Sepi, each 3 min.

##### Porosity

To calculate the porosity percentage (P), previously dried APM-Plur, APM-Carb, and APM-Sepi were immersed in absolute ethanol for 2 min and then weighed after removing excess ethanol from the surface with the help of a tissue. The porosity percentage was calculated using the following equation:

(3) $$P\;(\%)=\frac{W2-W1}{\rho \;\times \;V}\times 100$$

*W*1 represents the weight of the dried APM-Plur, APM-Carb, and APM-Sepi; *W*2 stands for the weight of APM-Plur, APM-Carb, and APM-Sepi after being immersed in ethanol; ρ is the density of absolute ethanol; and *V* is the volume of the gel.

##### Rheological Behavior

The rheological properties were assessed using a Thermo Scientific Haake Rheostress 1 rotational rheometer, which is manufactured by Thermo Fisher Scientific (Karlsruhe, Germany). The viscosity and flow curves were initially measured at a temperature of 25 °C for APM-Carb and APM-Sepi. For APM-Plur, the tests were carried out at 4 and 25 °C due to the thermoreversible characteristic of the pluronic gel. Test conditions included an acceleration period from 0 to 50 s^−1^ for 3 min, a constant shear rate of 50 s^−1^ for 1 min, and a decline period from 50 to 0 s^−1^ for 3 min. The data obtained from the flow curve (shear stress (τ) versus shear velocity (γ˙)) were fitted to the following mathematical models: Newton, Bingham, Ostwald-de-Waele, Herschel–Bulkley, Casson, and Cross. Likewise, the determination of the disturbance of the microstructure during the test, or “apparent thixotropy” (Pa/s), was evaluated by determining the area of the hysteresis loop. These tests were performed 24 h after the gels were prepared.

#### 2.2.3. Stability Studies

The three APM gels were evaluated for 60 days with regard to color, pH, homogeneity, consistency, and phase separation at 25 ± 2 °C/65 ± 5% RH.

#### 2.2.4. Release Profile

APM gel release studies were conducted using Franz vertical diffusion cells with a capacity of 13 mL (Franz Diffusion Cells 400; Crown Glass, Somerville, NJ, USA) and dialysis membranes with a molecular weight cutoff (MWCO) of 12 KDa. The membranes were hydrated for 24 h with a methanol/water mixture (50:50 *v*/*v*), washed with water, and then placed between the donor and receptor compartments. The effective diffusion area was 0.64 cm^2^. The receptor compartment was filled with Transcutol-P/water medium (60:40 *v*/*v*) and stirred at 700 rpm continuously. In the donor compartment, 80 mg samples of APM gels were added, and the procedure was maintained at 32 ± 0.5 °C to simulate in vivo skin conditions. At predetermined time intervals up to 74 h, 0.2 mL aliquots were withdrawn from the receptor compartment and replaced with an equal volume of the receptor medium. The concentration of APM released from the gels was quantified using HPLC [10]. The reported values represent the mean ± SD of five replicates. Various mathematical models were applied to fit the data and determine the release kinetics, with the model selected based on the correlation coefficient (*r*^2^).

#### 2.2.5. Skin Permeation Studies

In accordance with the Hospital de Barcelona Ethics Committee (dated 17 January 2020), this study utilized human skin obtained from a healthy 38-year-old woman who underwent an abdominal lipectomy at Hospital de Barcelona, SCIAS, Barcelona, Spain, with prior written consent. The skin sample integrity was assessed by measuring transepidermal water loss (TEWL) using the Tewameter TM 300 (Courage & Khazaka Electronics GmbH, Cologne, Germany), and only samples with TEWL findings below 10 g/m^2^/h were included [10]. To prepare the assay, skin samples of 0.4 mm thickness were obtained by cutting with an Aesculap GA 630 dermatome (Aesculap, Tuttlingen, Germany). These 0.4 mm thick skin samples were then placed between the donor and receptor compartments of Franz diffusion cells (6 mL) with a diffusion area measuring 0.64 cm^2^. The receptor medium, maintained at 32 ± 0.5 °C, consisted of a solution containing Transcutol-P and water in a 60:40 (*v*/*v*) ratio, and it was stirred at 700 rpm to ensure sink conditions. In the donor compartment, 80 mg samples of APM gels (1.5 mg/mL) were applied to the outer surface of the skin. At specific time intervals within a 30-h period, 0.2 mL aliquots were extracted from the receptor compartment and replaced with an equal volume of the receptor medium. The quantity of APM that permeated was determined using HPLC [10]. The reported values represent the mean ± SD of three replicate measurements.

##### Determination of the Amount of Drug Retention in the Skin

Ultrasound-assisted extraction was utilized to determine the amount of APM retained in the skin (Qret µg/g skin/cm^2^) at the end of the permeation study. Following the removal of skin samples from Franz diffusion cells, they were cleaned using gauze soaked in a 0.05% dodecyl sulfate solution and subsequently washed with distilled water. The skin permeation surface was then divided into sections, weighed, and submerged in 1 mL of ACN for 30 min using an ultrasonic bath [11]. After that, the solvent samples were subsequently filtered and quantified by HPLC [10].

#### 2.2.6. Cytotoxity Study in HaCaT Cells

Cell viability in response to APM gels was assessed using an MTT (3-(4,5-dimethylthiazol-2-yl)-2,5-diphenyltetrazolium bromide) assay. The immortalized keratinocyte cell line (HaCaT) was seeded at 2 × 10^5^ cells/mL in 96-well plates (Corning) and incubated at 37 °C with 5% CO_2_ for 24 h to allow for cell adhesion. Experiments were conducted when the cell confluence reached 80–90%.

The HaCaT cells were cultured in Dubelcco’s modified Eagle’s medium (DMEM) with high glucose content supplemented with 25 mM HEPES, 1% non-essential amino acids, 100 U/mL penicillin, 100 µg/mL streptomycin, and 10% heat-inactivated Fetal Bovine Serum (FBS). Different dilutions of APM gels (150, 30, 15, 3, 1.5, and 0.75 µg/mL) were analyzed separately to evaluate their independent effects on cell viability. After 24 h of incubation, the HaCaT cells were washed with 1% sterile PBS and treated with MTT solution (5 mg/mL) for 2 h at 37 °C. Subsequently, the medium was carefully aspirated, and 0.1 mL of 99% pure dimethyl sulfoxide (DMSO) was added to lyse the cells and dissolve the purple MTT crystals. The cell lysate was transferred to a fresh 96-well plate, and the absorbance was measured at 540/630 nm excitation/emission wavelengths using an Automatic Microplate Reader (Modulus Microplate Multicode Reader-Turner Biosystems, Sunnyvale, CA, USA). In parallel, a negative control consisting of untreated cells was included for comparison. The absorbance values were directly proportional to cell viability, and the percentage of cell viability was calculated using the following equation.

(4) $$Cell\;viability=\left[\frac{ABS\;treated\;cells}{ABS\;control\;cells}\right]\times 100$$

#### 2.2.7. Biomechanical Skin Properties

In vivo human skin tolerance studies focusing on evaluating biomechanical properties were carried out with the informed consent of the participating individuals. Total skin water loss (TEWL) was assessed using a Tewameter^®^ (TM 300, Courage-Khazaka Electronics GmbH, Cologne, Germany). This instrument measures the amount of water that diffuses and evaporates from the epidermal layer of the skin into the surrounding environment. Ten healthy participants were enlisted for the study and were given instructions not to apply any skincare cosmetics to the inner side of their left forearm for 24 h before the measurements. Prior to conducting the experiments, small circles were marked on the skin, and baseline measurements were recorded. Subsequently, a uniform layer of about 0.5 g of the blank gels (without APM) was applied to the center of the circle. Measurements were performed before applying the gels (basal readings) and 15 min, 1 h, and 2 h post-application. The probe of the Tewameter^®^ TM 300 (Courage-Khazaka Electronic GmbH, Cologne, Germany) was pressed against the designated area of the skin and held for 60 s to obtain the measurements. Skin temperature and stratum corneum hydration (SCH) were determined using a Corneometer^®^ 825, which was mounted on a Multi Probe Adapter^®^ MPA5 (Courage & Khazaka Electronics GmbH, Cologne, Germany). The measurement was performed by the capacitance method, which used the relatively high dielectric constant of water compared to the ones of other substances in the skin. Trans epidermal water loss (TEWL) data (expressed as g/h·m^2^), and all data were reported as the mean ± SD (n = 10).

#### 2.2.8. In Vivo Animal study: Imiquimod-Induced Psoriasis Skin Inflammation

The study protocol received approval from the Academic Ethics Committee of the Nursery of the Autonomous University of the State of Morelos, Mexico (protocol number: NOM-062-ZOO-1999).

The experiment aimed to assess the efficacy of APM gels after imiquimod-induced psoriasis skin inflammation in BALB/c mice aged 4–5 months (n = 6/group). The animals were housed in a temperature- and humidity-controlled room with ad libitum access to food and water. Hair on the dorsal region of animals was shaved using a trimmer 48 h before the initiation of the study. To group the animals, they were given a number from 1 to 30 and were divided into 5 groups (A–E). The numbers were randomized using an online random number generator (GIGAcalculator) [26]. To form the groups, the first 6 animals given by the random number generator were assigned to group A; the following 6 were assigned to group B; and so on. The experimental groups included the following: negative control (untreated animals), positive control (treated with imiquimod (IMIQ) only), and APM gels (APM-Carb gel, APM-Plur gel, and APM-Sepi gel).

**Figure 1:**
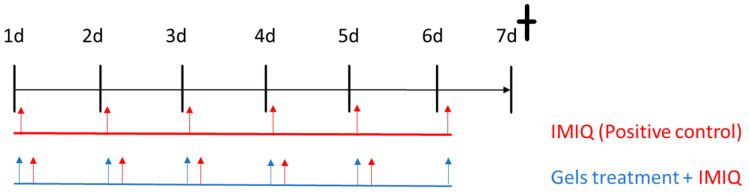
Plan study for IMIQ-induced skin inflammation and treatment with APM gels.

The psoriasis skin inflammation model was induced on the backs of the mice by applying 62.5 mg of IMIQ cream, Aldara^®^ (Meda Pharmaceuticals, Austria), in the mornings for five days in all groups, except the negative control. Application of 40 mg of freshly prepared APM gels (1.5 mg/mL) was made directly to the dorsal skin 6 h after IMIQ had been applied (see Figure 1). IMIQ and APM gel treatments were given for 5 days, while APM gels alone were applied one extra day. On day 7, the euthanasia of the animals was performed by cervical dislocation.

##### Score for Skin Inflammation

To assess the severity of inflammation on the back skin, this article has applied the method previously published [27]. The scoring system was inspired by the clinical Psoriasis Area and Severity Index (PASI), with slight modifications for the mouse model. Erythema, scaling, and thickening were evaluated independently, and each received a score on a scale from 0 to 3, where the symptoms were characterized as follows: 0: None; 1: Slight; 2: Moderate; 3: Severe. The cumulative score (erythema plus scaling plus thickening) served as a measure of the severity of inflammation (scale 0–9).

##### Histological Analysis

After the animal sacrifice, the back skin was removed, washed in PBS, and then treated with a 4% buffered paraformaldehyde solution overnight. The samples were then washed in PBS three times and dehydrated in a series of graded ethanol solutions (70%, 90%, and 100%). Following this, the samples were incubated in xylene and embedded in paraffin. The samples were then cut into 5 μm sections, stained with hematoxylin and eosin, and observed under a light microscope (Olympus BX41) equipped with an Olympus XC50 camera (Olympus Co., Tokyo, Japan). To serve as controls for the experiment, tissues without any formulations and tissues treated with imiquimod (induced skin inflammation) were used as the control and positive control conditions, respectively.

##### RNA Extraction and RT-qPCR Assays

Total RNA was extracted from small pieces of mouse skin back tissue using the RNeasy Mini Kit (Quiagen, Germantown, MD, USA) following the manufacturer’s instructions. Then, RNA was quantified using a NanoDrop spectrophotometer (Thermofisher, Waltham, MA, USA) and retro-transcribed into cDNA (complementary DeoxyriboNucleic Acid) to perform a qPCR reaction. For that, the SuperScript™ III Reverse Transcriptase kit and random primers were used, following the manufacturer’s instructions. After that, cDNAs were diluted at a ratio of 1:10 with distilled DNase/RNase-free water in a final volume of 200 μL. Samples were stored at −20 °C for long storage. To perform the qPCR reaction, PowerUp™ SYBR™ Green Master Mix was used, following the protocol recommended by the manufacturer using 96-well plates. Primers targeted different inflammatory genes, IL17A, IL17F, IL23, and IL8, and B-actin for a housekeeping gene (Table 1). qPCR reactions were carried out in a StepOnePlus™ thermocycler, and the cycle threshold (Ct) was calculated following the formula 2^−ΔΔCt^.

#### 2.2.9. Statistical Analysis

For the statistical analysis of the experimental data, GraphPad Prism v8.0 software (GraphPad Software Inc., San Diego, CA, USA) was used. The results are presented as the mean ± standard deviation (SD). To compare the mean values among different groups, a one-way analysis of variance (ANOVA) was applied. Subsequently, Tukey’s test was utilized for post hoc analysis to assess the specific differences between groups. Statistical significance was set at a *p*-value < 0.05. Any differences with a *p*-value below this threshold were regarded as statistically significant.

## 3. Results

### 3.1. Optimization and Preparation of APM Gels

Table 2 shows the physical aspects of the formulations obtained using different concentrations of Transcutol-P (20, 30, and 40%, *w*/*v*). Improved organoleptic properties were obtained when the formulations were prepared with 30% Transcutol-P. Therefore, this was selected as the optimal solvent concentration for the final formulations. Table 3 shows the composition of the APM gels.

**Figure 2:**
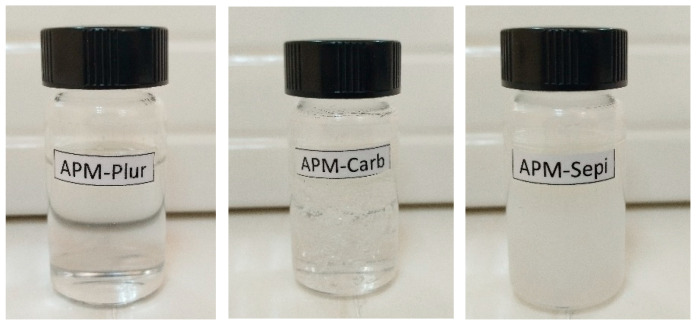
Physical aspects of APM-Plur, APM-Carb, and APM-Sepi.

APM-Plur, APM-Carb, and APM-Sepi show optimal organoleptic characteristics with excellent properties of homogeneity and consistency without the presence of lumps or precipitates (Figure 2).

### 3.2. Swelling, Degradation Tests, and Porosity

**Figure 3:**
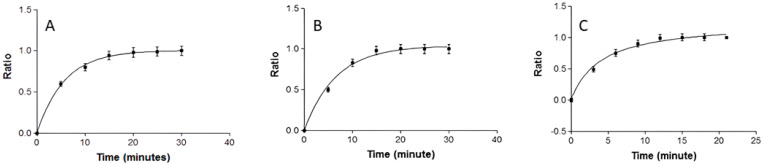
Swelling ratio of APM formulations evaluated at 32 °C in PBS at pH 5.5: (**A**) APM-Plur; (**B**) APM-Carb; (**C**) APM-Sepi.

**Figure 4:**
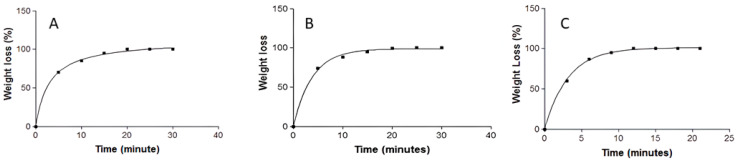
Degration profile of APM formulations evaluated at 32 °C in PBS at pH 5.5: (**A**) APM-Plur; (**B**) APM-Carb; (**C**) APM-Sepi.

The APM-Plur swelling process adhered to a first-order kinetic model, as depicted by the kinetic constant k = 0.17 min^−1^ (*r^2^* = 0.9995) (Figure 3). APM-Plur degradation was accomplished within 25 min, following a hyperbola model with a kinetic constant k = 0.27 min^−1^ (*r^2^* = 0.9994) (Figure 4), and the P percentage of APM-Plur was 89.74 ± 0.19%. The swelling process of APM-Carb followed a one-phase exponential model, represented by the kinetic constant k = 0.15 min^−1^ (*r^2^* = 0.9975) (Figure 3). The degradation of APM-Carb was completed in 20 min and followed a one-phase exponential model with a kinetic constant k = 0.26 min^−1^ (r^2^ = 0.9972) (Figure 4), and the P percentage of APM-Carb was 96.15 ± 0.16%. The swelling process of APM-Sepi followed a hyperbola model, represented by the kinetic constant k = 0.64 min^−1^ (*r^2^* = 0.9966) (Figure 3). The degradation of APM-Sepi was completed in 12 min and followed a one-phase exponential model with a kinetic constant k = 0.31 min^−1^ (*r^2^* = 0.9992) (Figure 4), and the P percentage of APM-Sepi was 86.32 ± 0.10%.

### 3.3. Rheologic Studies

**Figure 5:**
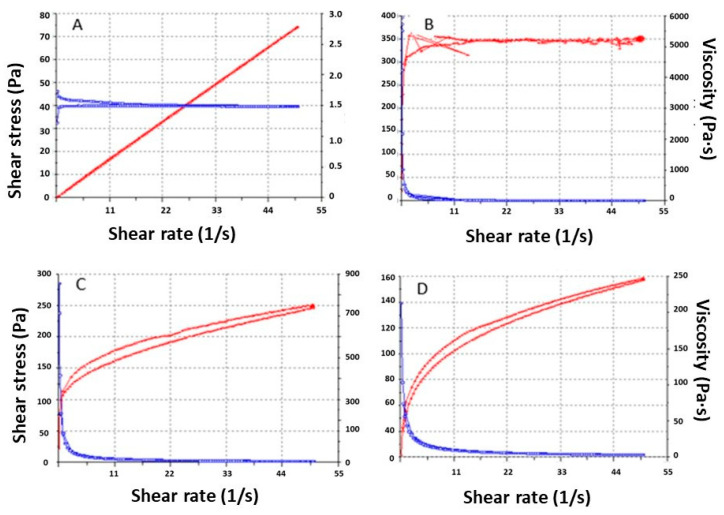
APM gel rheograms after 24 h: (**A**) APM-Plur at 4 °C; (**B**) APM-Plur at 25 °C; (**C**) APM-Carb at 25 °C; (**D**) APM-Sepi at 25 °C.

The rheology results at 4 and 25 °C are shown in Table 4 and Figure 5. The APM-Plur does not flow under the shear conditions of the experiment at 25 °C. All the formulations show thixotropy. APM-Plur and APM-Sepi show pseudoplastic behavior, contrary to APM-Carb, which shows plastic behavior.

### 3.4. Stability Studies

All the APM gels have maintained their physical characteristics, such as a white or transparent appearance and smooth and homogeneous consistency, and no phase separation was observed after 60 days of storage at 25 °C. Likewise, the pH levels stayed within the appropriate range for dermal administration, as indicated in Table 5.

### 3.5. Release Studies

**Figure 6:**
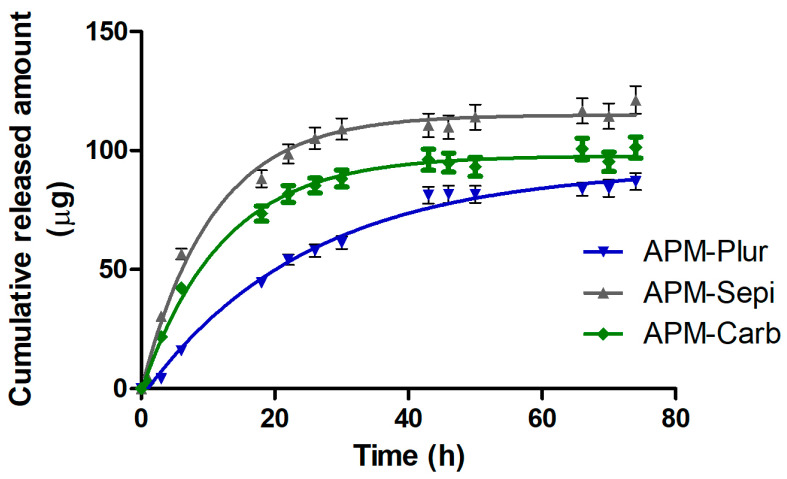
Release profile of APM gels.

The release patterns of APM from the gels (n = 5) are illustrated in Figure 6. The graphical representation of the cumulative released amount of APM against time reveals a Fickian passive diffusion model for all APM gels with a *r^2^* value of approximately 0.96 and a faster drug diffusion within the first 22 h, followed by a sustained release of the drug. This model suggests that the release of APM follows a first-order rate process, where the release rate is proportional to the remaining amount of the drug. After 74 h, a total of 121.24 µg of APM was released from Sepigel, followed by 101.26 µg of carbomer gel and 86.98 µg from pluronic gel. Consequently, the APM-Sepi shows a higher AUC (see Table 6). The maximum release amount (Ymax) of APM from Sepigel was estimated to be 115.00 µg, and the release constant (K) was calculated to be 0.094 h^−1^.

There are no overlapping curves in the models, showing significant differences between all APM gels (see Table 7).

### 3.6. Skin Permeation and Retained Amount on the Skin

After conducting the permeation test for 30 h using the APM gel (n = 3) formulations, APM was not detected in the receptor fluid. However, upon extracting the skin samples, the presence of APM was observed, with a quantity of the drug retained in the skin (*Qret*) of 385.54 ± 102.56 µg for APM-Plur, 393.13 ± 115.60 µg for APM-Carb, and 401.56± 27.32 µg for APM-Sepi per gram of skin per square centimeter (µg APM/g skin/cm^2^).

### 3.7. In Vitro Studies: Cytotoxicity Data

**Figure 7:**
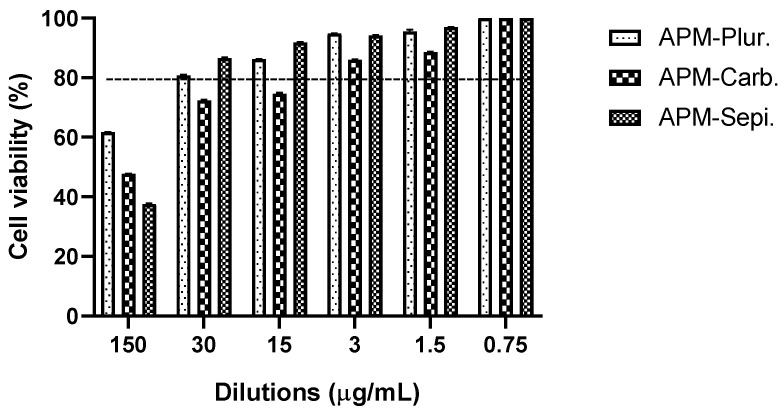
Cytotoxicity assay for APM gels.

In the in vitro cytotoxicity assay using HaCaT cells, the 3-(4,5-dimethylthiazol-2-yl)-2,5-diphenyltetrazolium bromide (MTT) method was employed. The cells were exposed to APM gels at six different concentrations. The results of the assay indicate that cell viability remained greater than 80% in the tested dilutions, ranging from 0.0005 to 0.02 mg/mL (Figure 7). The APM-Carb was the most tendentious to have lower values for the viability; instead, the APM-Plur and APM-Sepi show greater values. These data suggest that APM gels did not significantly impact the viability of HaCaT cells, indicating their potential safety for use in the tested cellular context.

### 3.8. In Vivo Tolerance Studies: Biomechanical Skin Properties

**Figure 8:**
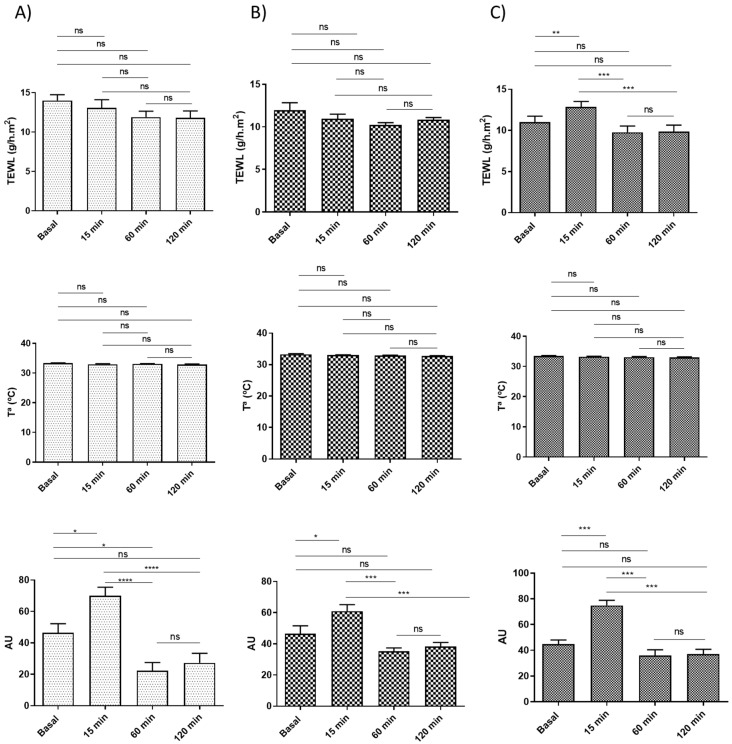
Biomechanical parameter evolution monitored before the application of the formulations and 15 min, 1 h, and 2 h after application: (**A**) Blank-Plur; (**B**) Blank-Carb; (**C**) Blank-Sepi. TEWL is expressed as g/h × cm^2^, SCH as arbitrary units (AU), and temperature as degrees Celsius (°C). Significant statistical differences: * *p* < 0.03, ** *p* < 0.002, *** *p* < 0.0002, **** *p* < 0.0001, ns = non-significant.

Figure 8 shows the changes in monitored biomechanical parameters before and after the application of the blank gels for 120 min. Regarding transepidermal water loss (TEWL) values, no statistically significant differences were observed on the volunteers’ skin after applying Blank-Plur and Blank-Carb with respect to the basal state (Figure 8A,B). However, it was possible to observe a little increase in the TEWL value at 15 min after application of Blank-Sepi, followed by a decrease at 60 min post-application, and it remained stable thereafter (Figure 8C).

No changes in skin temperature were demonstrated for any of the gels, as can be observed in Figure 8A–C.

The stratum corneum hydration (SCH) results obtained from Blank-Plur indicate an increase 15 min after application, followed by a decrease at 60 min and a little increase at 120 min (Figure 8A). For Blank-Carb, significant differences were observed at 15 min and gradually rose until reaching a stable level (Figure 8B). On the other hand, SCH values obtained from Blank-Sepi displayed a similar profile to those obtained from Blank-Carb, increasing the values 15 min after application and remaining unchanged after 60 min (Figure 8C).

### 3.9. In Vivo Animal Study: Imiquimod-Induced Psoriasis Skin Inflammation

**Figure 9:**
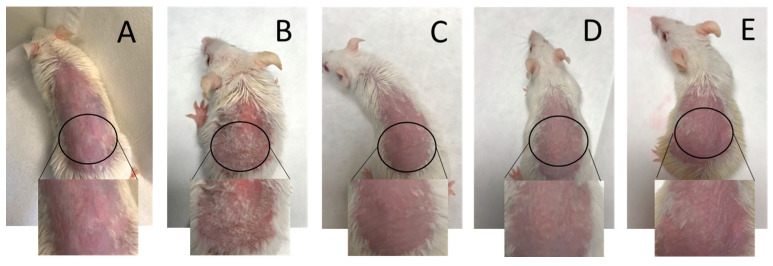
Back skin treatment after 6 days: (**A**) negative control; (**B**) positive control; (**C**) APM-Sepi; (**D**) APM-Plur; (**E**) APM-Carb.

The efficacy studies using imiquimod-induced psoriasis skin inflammation in BALB/c mice were evaluated. APM gels were administered as a follow-up treatment after a 6-h interval following the daily application of IMIQ for 5 days in a row. On the second day of treatment, the back skin of the mice started showing signs of erythema (Figure 9).

#### 3.9.1. Score for Skin Inflammation

**Figure 10:**
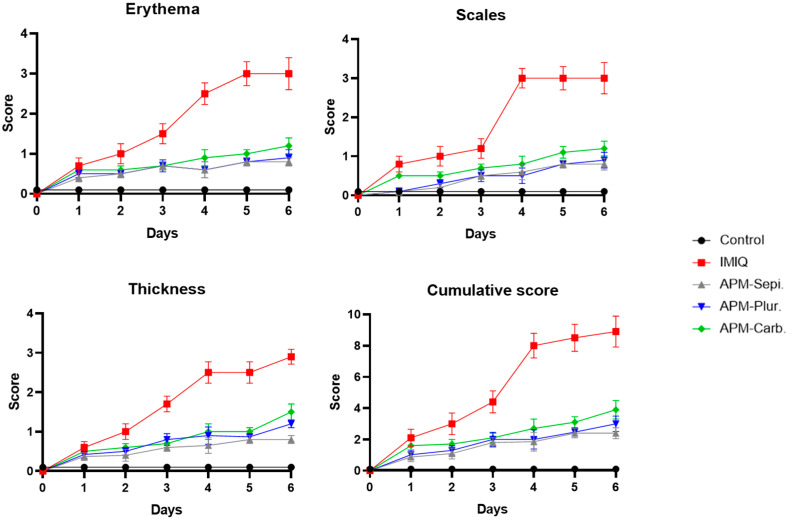
Erythema, scaling, and thickness of the back skin on a scale from 0 to 3 on a daily basis. The cumulative score (erythema plus scaling plus thickening) on a scale from 0 to 9. Mean score ± SD (n = 6/group).

To investigate whether topical application of IMIQ induces skin inflammation, a skin score was determined. IMIQ cream was applied to the shaved back skin of the mice for six consecutive days. Signs of erythema, scaling, and thickening began to appear on the back skin of the mice. These inflammatory features progressively intensified throughout the experiment and were represented by independent scores (Figure 10). Notably, the group control did not display any signs of inflammation. Moreover, the cumulative score showed a significant reduction using APM gels compared to IMIQ only.

#### 3.9.2. Histological Analysis

**Figure 11:**
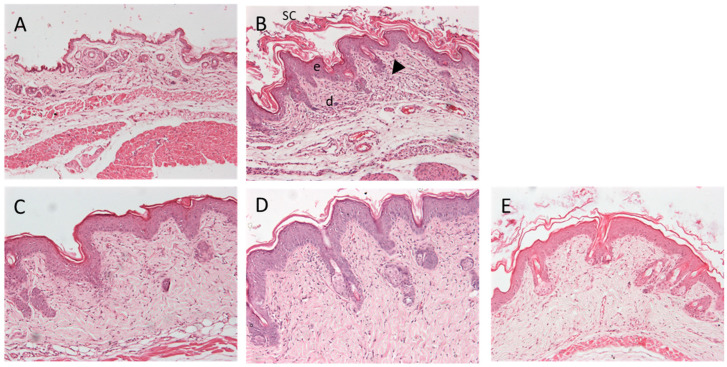
Macroscopic presentation of the back skin of the mice. (**A**) Negative Control; (**B**) Positive Control; (**C**) APM-Sepi; (**D**) APM-Plur; (**E**) APM-Carb. The arrowhead indicates leukocyte infiltration. SC = stratum corneum, e = epidermis, d = dermis. Images at 100× magnification. Scale bar = 200 µm.

The treatment with IMIQ induced a psoriasis-like skin inflammation that could be appreciated in the histology of the back skin by means of an increase in the epithelial cell thickness and in leucocyte infiltrate (Figure 11B). The treatment with the different formulations was able to reduce the leucocyte infiltrate. However, no effect was observed in the epithelium thickness, even though APM-Sepi (Figure 11C) and APM-Plur (Figure 11D) were able to reduce the uppermost layer of the skin, made up of dead keratinocytes of the stratum corneum.

#### 3.9.3. RNA Extraction and RT-qPCR Assays

**Figure 12:**
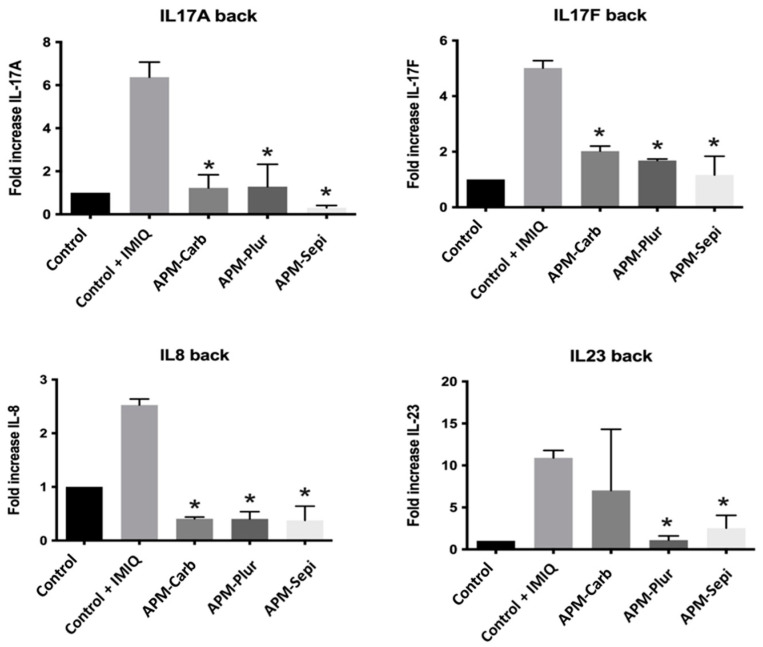
qPCR analysis of the back skin of the mice after the treatment with APM gels. Significant statistical differences: * *p* < 0.05.

Results from qPCR show that the IMIQ conditions used to induce the psoriasis-like model were positive for inflammation induction, as all inflammatory cytokines (IL-17A, IL-17, IL-8, and IL-23) tested were significantly upregulated. The application of IMIQ to mouse skin leads to the recruitment of various immune cells and the enlargement of the epidermal layer (hyperplasia). Regarding the different treatments tested, Figure 12 shows that the three gels (APM-Carb, APM-Plur, and APM-Sepi) have an anti-inflammatory effect, reducing the four cytokines used in this experiment. However, it seems that the two best are APM-Sepi and APM-Plur, as in IL-17A, IL-17F, and IL-23, the inflammation reduction is bigger compared to APM-Carb.

## 4. Discussion

Psoriasis is a chronic inflammatory skin disease characterized by well-defined, scaly, red lesions, most frequently located on the elbows, knees, scalp, hands, and feet. This disease can affect the quality of life of people since patients can suffer significant physical discomfort, disability, itching, and pain. They can interfere with basic functions, such as self-care or sleep. Skin lesions on the hands can prevent certain jobs, sports activities, or caring for relatives at home [28].

In this study, three apremilast-loaded gels, APM-Plur, APM-Carb, and APM-Sepi, were developed to treat psoriasis topically were developed. These gels showed optimal organoleptic characteristics for dermal application, with excellent properties of homogeneity and consistency, being pleasant to the touch and easy to use. These gels were prepared using 30% Transcutol-P, which is an ethylene oxide derivative that, thanks to its strong solubilizing potential coupled with its low toxicity and high biocompatibility, is widely used as a solvent in a variety of products, including pharmaceuticals and nutraceutical products, dietary supplements, cosmetics, and food additives [29].

The pH values were 5.5, 6.3, and 5.3 for APM-Plur, APM-Carb, and APM-Sepi, respectively. This result confirms the biocompatibility of the three formulations with the skin and thus guarantees non-irritating effects. On the other hand, the swelling capacity, which measures the amount of liquid that the gel can absorb inside, was analyzed. The swelling kinetic profiles were first-order kinetic models for APM-Plur and APM-Carb and the hyperbola model for APM-Sepi, whereas their respective degradation processes were completed in 25, 20, and 12 min. The porosity percentage of the three gels was higher than 85%, which indicates that the drug could be released through the pores formed by these polymers to exert the desired effect. Previous studies have shown a similar degradation process with rapid degradation and a high porosity percentage [20,30,31].

The rheological analysis provides information about ease of administration, sensory properties, fill/dispense characteristics, and modulates biopharmaceutical parameters such as drug release rates from its vehicle [31]. APM-Plur exhibits pseudoplastic behavior and an average viscosity of 1503 ± 0.97 mPa·s at 4 °C. This result differs from other studies where pluronic gels show lower viscosity values at low temperatures [31]. The high viscosity of APM-Plur was also observed at 32 °C. This behavior could be due to the content of Transcutol-P, since this excipient increases the viscosity of gel formulations.. In the case of APM-Carb, it showed a plastic behavior and an average viscosity of 5097 ± 28.83 mPa·s. Other studies with carbomer gels at 1% have shown a pseudoplastic behavior that differs from our results, possibly due to the concentration of the polymer since in the present study 2% carbopol was used [32]. Finally, APM-Sepi showed an average viscosity of 3086 ± 5.50 mPa·s and pseudoplastic behavior, which is characteristic of this type of gels.. Additionally, the three formulations showed a hysteresis loop in the rheograms, indicating a thixotropic behavior of the gels. The thixotropic behavior of topical application formulations favors their dermal administration since the viscosity of the formulation is reduced with the friction with which they are applied, facilitating their spreadability, and is returned to the initial viscosity once the friction stops, easing their retention on the skin [31].

The release evaluation of a drug from its vehicle is part of the quality control studies of pharmaceuticals. This parameter is determined by interactions between the drug and its vehicle, which in turn define its bioavailability and thus its efficacy [33]. In this research, the three formulations released the contained drug over 74 h, although a higher release rate was observed in APM-Sepi (115.00 µg) compared to APM-Plur (92.56 µg) and APM-Carb (97.84 µg). The release profiles of APM from the gels followed a first-order model (Fickian kinetic), indicating that the release rate is directly proportional to the concentration of drug remaining in the vehicle [10]. Additionally, there was a similarity between the degradation time and the drug release, resulting in the shorter degradation time of the gel matrix, the higher the drug release. The correlation between gel degradation and drug release is typically observed because the degradation of the gel or polymer matrix can influence the rate and mechanism of drug release [34].

The efficacy of topical dermal treatments relies on the drug’s capability to penetrate the stratum corneum and diffuse across the epidermis and dermis [35]. Ex vivo permeation studies using human skin are employed to assess this ability and forecast the in vivo behavior of dermal formulations [36]. The results of this study revealed that the APM penetrates through the stratum corneum without reaching the receptor fluid in either of the three formulations, but this drug is retained in the skin, thereby it could be used as an effective local treatment for skin inflammation without systemic adverse effects. All gels showed a high amount of retained drug in the skin; however, a slightly higher result was observed in APM-Sepi (401.56 ± 127.32 µg APM/g skin/cm^2^) compared to APM-Plur (385.54 ± 102.56 µg APM/g skin/cm^2^) and APM-Carb (393.13 ± 115.60 µg APM/g skin/cm^2^). This result could be due to the nature of the Sepigel that constitutes polyacrylamide-based emulsion that make it a multifunctional vehicle that improves the tissue-adhering properties of topical formulations favoring the drug permeation [37].

The tolerance for topical administration on the skin of the three formulations was evaluated by in vitro and in vivo models. The HaCaT cell line is widely used for in vitro evaluation of the toxicity of dermal formulations before conducting pre-clinical assays using animal models. This is because, like most cell lines, HaCaT cells are easy to cultivate, exhibit rapid growth, and display high sensitivity to toxic stimuli [36]. In this work, the results confirmed that the three APM gels do not induce relevant cytotoxic effects in the HaCaT cells, indicating their adequate biocompatibility with human keratinocytes, especially for APM-Plur and APM-Sepi, which showed greater viability values. These results were supported by further studies in volunteers on the evaluation of biomechanical skin properties, including transepidermal water loss (TEWL), temperature, and stratum corneum hydration (SCH), before and after the application of lank APM gels. These parameters are evaluated in dermatology to detect skin barrier irritation after exposure to physical or chemical agents in the early stages. Disturbances in the integrity of the skin cause an increase in TEWL and a decrease in SCH, producing dryness, cracks, and fissures in the stratum corneum [38]. Blank-Plur and Blank-Carb did not induce notable differences in TEWL value following skin application when compared to the baseline condition. However, a significant TEWL increase was observed after 15 min of applying Blank-Sepi, although it exhibited a tendency to return to the baseline state after 1 h. On the other hand, the three gels caused a significant increase in SCH values after 15 min of skin application and a return to the basal state without significant differences after 120 min. Additionally, no changes in skin temperature were observed during the experiment for any of the three formulations. The observed slight decrease in hydration after 60 min of skin application can be explained by the gel’s capacity to absorb and retain moisture from the skin, leading to a temporary reduction in SCH values. However, at 120 min, the gel likely reached a saturation point, resulting in stabilization and a lack of significant differences in SCH values compared to the basal values. It is noteworthy that no visible signs of skin irritation were detected in volunteers following the dermal application of blank gels. This demonstrates that the gels were well-tolerated by the skin and did not cause any significant irritation.

Regarding anti-inflammatory efficacy, this research aimed to determine if imiquimod-induced inflammation in mice could serve as a reliable model for studying human psoriasis. Imiquimod is a toll-like receptor (TLR) 7 agonist that is widely utilized in mouse models of psoriasis-like skin inflammation since topical imiquimod application onto the mouse ear or back skin produces a phenotype associated with many human psoriasis characteristics, including erythema, scaling, edema, and increased epidermal production of IL-23, 17A, and 17F [39,40]. Moreover, imiquimod as a model of psoriasis offers ease of use, the acute nature of the inflammatory response, convenience, and low cost [39]. In this study, APM gels reduced the cumulative score of signs of erythema, scaling, and thickening on the back skin of mice (Figure 10). In the same way, the histological analysis revealed the potential of the APM formulations to reduce the leucocyte infiltrate in the treated area. APM-Sepi and APM-Plur showed a special role in reducing the uppermost layer of the skin formed by dead keratinocytes. These results were consistent with those obtained in the biochemical studies, which confirmed that the three APM gels decreased the overexpression of all the proinflammatory cytokines studied. APM-Sepi showed a greater decrease in the expression of IL17A, IL17F, and IL-8, whereas APM-Plur IL-23 exhibit greater efficacy in reducing IL-23. These results hold significant importance because the clinically relevant signaling pathways in psoriasis are primarily mediated by IL-17A and IL-17F. Both of these cytokines act through the same receptor, but exhibit varying degrees of potency. IL-17A tends to exert a stronger effect than IL-17F, and it is precisely where a better effect is seen by the APM gels tested [41]. Likewise, the three APM gels showed a marked decrease in the production of IL-8, which has been implicated in the early stages of the pathogenesis of psoriasis. Regarding IL-23, its expression is increased in skin lesions. Specifically, drugs directed against IL-23 and IL-17 are effective in the clinical treatment of plaque psoriasis [42]. Taken together, these results suggest the promising benefits of APM gels, mainly APM-Sepi and APM-Plur, in the topical treatment of psoriasis, making them a potentially attractive option for further development and clinical application.

## 5. Conclusions

The findings from this study provide strong support for the topical application of APM gels as an effective therapeutic strategy in the treatment of psoriasis skin inflammation. The APM gels were shown to be homogeneous and white or transparent. A high tolerability of blank gels in healthy volunteers is an advantage, attributed to their composition based on approved excipients known for their high biocompatibility with the skin. This ensures a reduced risk of adverse reactions when applied to the skin. The anti-inflammatory potential of APM gels is evidenced by various findings. In vitro experiments showed a reduction in the production of the inflammatory cytokines IL-17 and IL-23, especially for APM-Sepi and APM-Plur. In summary, these results collectively highlight the promising therapeutic benefits of APM gels, with significant improvement in psoriasis-like skin induced by IMIQ, making it a potentially attractive option for further development and clinical application.

## Figures and Tables

**Table 1:** Gene-specific primers utilized in real-time qPCR.

Gene	Primer Sequence (5′ to 3′)
GAPDH	FW: AGCTTGTCATCAACGGGAAG
	RV: TTTGATGTTAGTGGGGTCTCG
IL-8	FW: GCTGTGACCCTCTCTGTGAAG
	RV: CAAACTCCATCTTGTTGTGTC
IL-23	FW: GAGCCTTCTCTGCTCCCTGATA
	RV: GACTGAGGCTTGGAATCTGCTG
IL-17A	FW: TTTTCAGCAAGGAATGTGGA
	RV: TTCATTGTGGAGGGCAGAC
IL-17F	FW: TTCCAAAAGC CTGAGAGTTG
	RV: GCCCAAGTTC CTACACTGG
TNFα	FW: AACTAGTGGTGCCAGCCGAT
	RV: CTTCACAGAGCAATGACTCC

GAPDH = glyceraldehyde-3-phosphate dehydrogenase; IL-8 = interleukin-8; IL-23 = interleukin-23; IL-17A = interleukin-17A; IL-17F = interleukin-17F; TNFα = tumor necrosis factor alpha; FW = forward primer; and RV = reverse primer.

**Table 2:** Physical aspects of APM gels at different concentrations of Transcutol-P.

	Transcutol-P Concentration (%)	Color	Homogeneity	Consistency	Phase Separation
	20	Turbid	++	++	None
APM-Plur	30	Transp.	+++	+++	None
	40	Transp.	+++	++	None
	20	White	++	+	None
APM-Carb	30	White	+++	+++	None
	40	White	++	++	None
	20	White	++	+	None
APM-Sepi	30	White	+++	+++	None
	40	White	++	++	None

The APM-Plur gel was evaluated at 25 °C. Transp. = Transparent. +++ (excellent); ++ (good); + (fair).

**Table 3:** Composition of the final formulations of the apremilast gels.

Components (%, *w*/*v*)	APM-Plur	APM-Carb	APM-Sepi
Apremilast (APM)	0.15	0.15	0.15
Transcutol-P	30	30	30
Hyaluronic acid (HA)	0.2	0.2	0.2
Pluronic F 127	20	-	-
Carbomer 940	-	2	-
NaOH 1N		0.4	
Sepigel 305^®^	-	-	2
MQ Water	49.65	67.25	67.65

**Table 4:** Results obtained from the rheological study of APM gels.

Formulation	Viscosity at 4 °C mPa·S	Viscosity at 25 °C mPa·S	Thixotropy Pa/s	Rheological Behavior (Best Equation)
APM-Plur	1503.00 ± 0.97	The product does not flow under the shear conditions of the experiment.	36.62	Pseudoplastic (Cross model; r^2^ = 1)
APM-Carb	NA	5097.00 ± 28.83	2093.00	Plastic (Herschel–Bulkley)
APM-Sepi	NA	3086.00 ± 5.50	411.50	Pseudoplastic (Cross model; r^2^ = 0.9998)

NA = not applicable.

**Table 5:** Stability studies of apremilast gels stored at 25 ± 2 °C/65 ± 5% RH for 60 days.

	Days	pH	Color	Homogeneity	Consistency	Phase Separation
APM-Plur	1	5.5 ± 0.08	Transp.	+++	+++	None
	60	5.3 ± 0.06	Transp.	+++	+++	None
APM-Carb	1	6.3 ± 0.07	White	+++	+++	None
	60	6.4 ± 0.06	White	+++	+++	None
APM-Sepi	1	5.3 ± 0.02	White	+++	+++	None
	60	5. 5 ± 0.03	White	+++	+++	None

Transp. = Transparent; +++ (excellent).

**Table 6:** Release parameters for APM gels.

	APM-Plur			APM-Sepi			APM-Carb		
Mean	SD		n	Mean	SD	n	Mean	SD	n
Plateau (µg)	92.56	5.69	5	115.00	4.03	5	97.84	3.42	5
K (1/h)	0.041	0.007	5	0.094	0.018	5	0.083	0.014	5
Half-time (h)	17.04	0.04	5	7.37	0.09	5	8.38	0.08	5
AUC (µg·h)	4557	471	5	7232	798	5	6004	599	5
*r* ^2^	0.9617			0.9533			0.9606		

**Table 7:** Statistical analysis.

Parameters	Plateau	K	Half-Time	AUC
Tukey’s Multiple Comparison Test	*p*-Value	*p*-Value	*p*-Value	*p*-Value
APM-Plur vs. APM-Sepi	***	***	***	***
APM-Plur vs. APM-Carb	ns	**	***	**
APM-Sepi vs. APM-Carb	***	ns	***	*

Where Plateau is the Y value at infinite times; K is the rate constant; and Half-time is in the time units of the X axis. Span is the difference between Y0 and Plateau. AUC is the area under the curve. * = *p* < 0.05; ** = *p* < 0.001; *** = *p* < 0.0001; and ns = non-statistically significant.

## Data Availability

The data presented in this study are available on request from the corresponding author.
